# Circulating miR-18b-3p is a novel biomarker predicting chemo-radiotherapy induced oral mucositis in head and neck cancer

**DOI:** 10.1038/s41392-025-02478-3

**Published:** 2025-11-21

**Authors:** Claudio Pulito, Renata Brandi, Rosanna Sestito, Giulia Orlandi, Giuseppe Sanguineti, Giovanni Blandino, Sabrina Strano

**Affiliations:** 1 IRCCS Regina Elena National Cancer Institute, Rome, Italy; 2 IRCCS San Gallicano Dermatological Institute, Rome, Italy

**Keywords:** Predictive markers, Inflammation

Dear Editor,

Head and neck squamous cell carcinoma (HNSCC), the sixth leading cancer worldwide, arises in the lining mucosa of the oral cavity, pharynx and larynx. Standard treatments such as surgery, radiation and chemotherapy are associated with major morbidity and toxicity. Five-years survival rates are rather poor. Oral mucositis (OM) is a painful, dose-limiting side effect affecting up to 90% of radio-chemotherapy treated head and neck squamous cell carcinoma (HNSCC) patients. The pathophysiology of OM is a multistage process which occurs across five phases: (1) initiation; (2) upregulation of inflammation; (3) signaling amplification; (4) ulceration and (5) wound healing. Despite its prevalence and clinical burden, no reliable biomarkers are currently available for either predicting or monitoring OM.^1^ MicroRNAs are highly stable tissue and body-fluid circulating small non-coding RNAs (19–24 nucleotides) originating from precursors RNAs that are involved in post-transcriptional regulation of coding genes. Their regulatory functions highlight a pivotal role in tumor development and tumor-associated diseases.^2^ In the present work the profiling of microRNAs released into the extracellular environment of human primary gingival epithelial cells (EGKs) treated with four distinct groups of anticancer drugs unveiled miR-18b-3p as that upregulated independently from the type of the anticancer treatment. These results were consistent across primary and immortalized gingival cell lines (IGK). miR-18b-3p release increased at sub-apoptotic dose-dependent treatment. It has been reported that radiotherapy and chemotherapy independently contribute to acute mucosal toxicity. When combined, these treatments increase the risk of high-grade oral mucositis by fourfold compared to radiotherapy alone.^3^ Indeed, we found that combined treatment of EGKs cells with a fixed amount of cisplatin and increasing radiation doses led to a greater increase in miR-18b-3p release than each treatment alone, suggesting either additive or synergistic effects (Fig. 1a). To strength the translational relevance of our pre-clinical findings we investigated circulating amount of miR-18b-3p in the plasma of 23 high-grade (stage III/IV) HNSCC patients undergoing conventional chemo-radiotherapy (RT-CT) and with clinical evidence of OM (Grade 2-3, additional file). For each of the 23 analyzed patients we assessed plasma circulating level miR-18b-3p at the baseline, at one month and at the end of the treatment. miR-18b-3p circulating amounts were significantly increased both after one month and at the end of treatment compared to baseline levels (Fig. 1b). One critical aspect of circulating analytes in any liquid biopsy approach resides on the specificity of the cellular source which releases either nucleic acids or proteins. Notably, increased amount of blood circulating miR-18b-3p paired with larger areas of irradiated tissues of the analyzed HNSCC patients (Fig. 1b). These findings strongly indicated that miR-18b-3p was mostly released by peritumoral and non-tumoral tissues than tumoral ones. In contrast, in a cohort of 10 HNSCC patients treated with stereotactic body radiation therapy (SBRT), a precision modality that spares non-tumoral tissues, with no clinical evidence of OM (additional file), no significant change in circulating miR-18b-3p was observed between the baseline and the end of treatment (Fig. 1b). Further supporting the link between miR-18b-3p release and mucosal injury. To decipher mechanistically the contribution of miR-18-3p to OM we initially evaluated the paracrine effect of released miR-18b-3p on endothelial cells, given their role in the early inflammatory and angiogenic phases of OM. We found that conditioned medium from cisplatin-treated EGKs significantly increased capillary-like structure formation in HUVECs, as reflected by both tube length and number of segments (Fig. 1c). With the due limitation of higher miRNA copies compared to those evidenced in conditioned cell medium and circulating in HNSCC patients with OM, overexpression of miR-18b-3p in HUVECs recapitulated this pro-angiogenic phenotype (Fig. 1c). These findings might suggest that the initial OM lesion is a receptacle of paracrine and autocrine factors among which microRNAs might confer specificity in activating or inhibiting specific pathways due to their ability to selectively targeting 3’-UTRs of specific mRNAs. Indeed, we performed bulk RNA-seq analysis of HUVECs overexpressing miR-18b-3p to identify its target mRNAs. Gene Set ranking of the differentially expressed genes comparing HUVEC-miR-18b-3p mimic and HUVEC-cntrl mimic revealed that genes belonging to cell cycle regulation, mitosis, G2M checkpoints were specifically enriched. We also identified transcripts that were significantly de-regulated in the two analyzed cell populations. Notably, the comparison between predicted miR-18b-3p targets and transcripts significantly downregulated in HUVEC-miR-18b-3p cells revealed a shared subset of genes, including SEMA3G and FOXJ2. These two transcripts, known inhibitors of endothelial cell proliferation and angiogenesis,^4,5^ were selected for further investigation. mRNA and protein expression analysis confirmed significant repression of both genes, and luciferase reporter assays using wild-type and mutated 3′UTRs of either SEMA3G or FOXJ2 transcripts demonstrated direct targeting by miR-18b-3p (Fig. 1d). Functionally, depletion of either SEMA3G or FOXJ2 expression recapitulated the pro-proliferative effects of miR-18b-3p in endothelial cells (Fig. 1d). These findings might indicate that miR-18b-3p stimulates HUVEC cell proliferation, at least in part, by impairing the anti-proliferative effects of FOXJ2 and SEMA3G genes. Indeed, we also provide evidence that miR-18b-3p either alone or in cooperation with pro-inflammatory cytokines released by the injured mucosal cells reshapes the OM cellular landscape promoting endothelial cell proliferation and vessel formation. These findings originally show that a synergistic crosstalk between microRNAs and other factors present in the OM receptacle might sustain the instauration and expansion of the mucosal lesion.

**Fig. 1:**
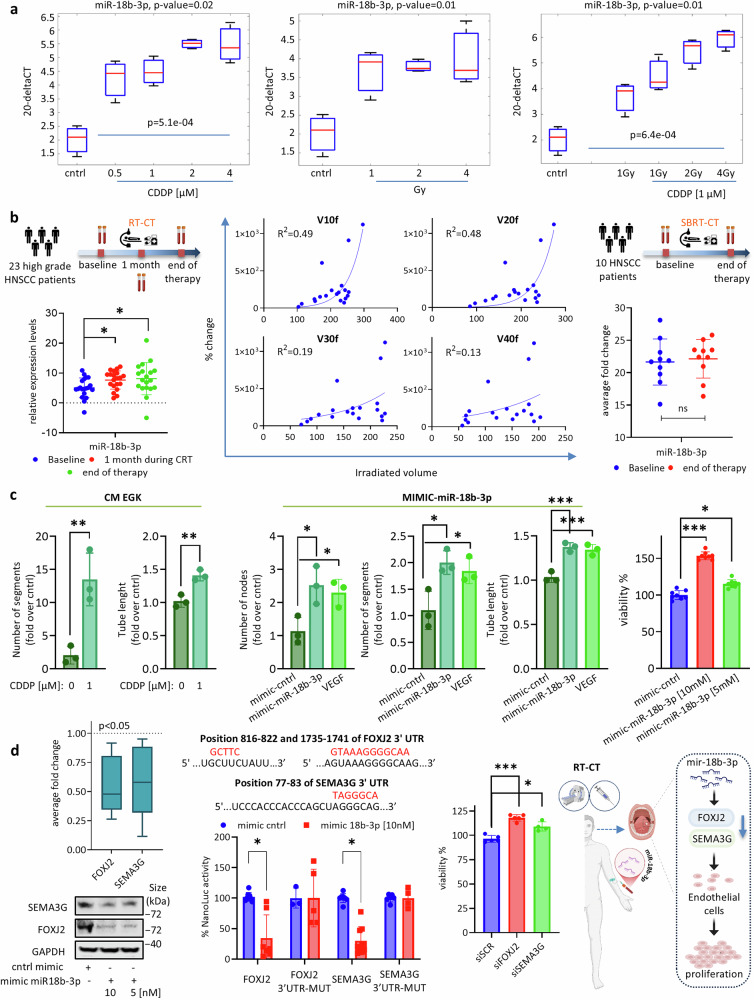
miR-18b-3p is a mechanistic determinant of anticancer drug-induced oral mucositis (OM) onset. **a** Boxplots showing 20^−ΔCT^ expression levels of miR-18b-3p measured in the media of IGK cells either untreated or treated with cisplatin (CDDP, left panel), radiotherapy (middle panel), or combined chemo-radiotherapy (right panel). Data are presented as the mean +/− SEM of at least five independent experiments. **b** Schematic overview of a clinical case study based on a retrospective cohort of 23 high-grade (stage III/IV) HNSCC patients treated with chemotherapy and intensity-modulated radiation therapy (IMRT) who developed OM (upper left panel). Plasma samples were collected at baseline, after one month, and at the end of therapy. Changes in circulating miR-18b-3p levels were measured relative to baseline (lower left panel). Statistical analysis: unpaired *t*-test, *p ≤ 0.05. The association between radiation dose to the oral mucosa and changes in circulating miR-18b-3p at the end of treatment was assessed (middle four panels). The x-axis represents the volume of oral mucosa exposed to a given radiation dose (Gy), and the y-axis shows the percentage change in miR-18b-3p expression. A second case study (upper right panel) included 10 HNSCC patients treated with stereotactic body radiotherapy combined with chemotherapy (SBRT-CT). Plasma samples were collected at baseline and at treatment completion; changes in circulating miR-18b-3p levels are shown (lower right panel). Statistical analysis: unpaired *t*-test, *p ≤ 0.05. **c** Histograms showing the number of segments and total tube length formed by HUVEC cells cultured in conditioned media from primary gingival keratinocytes (CM-EGK) treated for 72 h with the indicated cisplatin doses (first two panels, left). Data are presented as the mean +/− SEM of at three independent experiments. Statistical analysis: *t*-test,*p ≤ 0.05, **p ≤ 0.01, ***p ≤ 0.001. Additional histograms (middle panels) show the number of nodes, segments, and total tube length in HUVEC cells transfected with either a control mimic, miR-18b-3p mimic, or stimulated with VEGF. Data are presented as the mean +/− SEM of at three independent experiments. Statistical analysis: *t*-test,*p ≤ 0.05, **p ≤ 0.01, ***p ≤ 0.001. The rightmost panel shows HUVEC viability after transfection with control or miR-18b-3p mimics (5 and 10 nM). Data are presented as the mean +/− SEM of at least eitght independent experiments. Statistical analysis: *t*-test, *p ≤ 0.05, ***p ≤ 0.001. **d** Average fold change in the expression levels of SEMA3G and FOXJ2 relative to mimic control (dashed line), as evaluated by qRT-PCR in HUVEC cells overexpressing mimic miR-18b-3p (5 nM) (upper left panel). Statistical analysis: *p* ≤ 0.05. Representative western blot of whole-cell lysates from HUVEC cells overexpressing either mimic control or mimic miR-18b-3p (5 and 10 nM), stained with the indicated antibodies (lower left panel). The seed sequences of miR-18b-3p on the 3’UTRs of SEMA3G and FOXJ2 are shown (middle upper left panel), with mutated sequences in red. Histograms (middle lower left panel) report luciferase activity (as percentage) of NanoLUC-3’UTR constructs (wild-type and mutant) for SEMA3G and FOXJ2 in HEK293 cells transfected with control or miR-18b-3p mimics. Data are presented as the mean +/− SEM of at least three independent experiments. Statistical analysis: *t* test, *p ≤ 0.05. The middle right panel shows HUVEC viability after gene silencing with a control siRNA (siSCR), or a siRNA of FOXJ2 (siFOXJ2), or a siRNA of SEMA3G (siSEMA3G). Data are presented as the mean +/− SEM of at least five independent experiments. Statistical analysis: *t*-test, *p ≤ 0.05, **p ≤ 0.001. Schematic representation of the proposed model (final panel): miR-18b-3p functions as a mechanistic driver of OM and a potential circulating biomarker. At the paracrine level, it promotes HUVEC proliferation by inhibiting the anti-proliferative actions of FOXJ2 and SEMA3G

In aggregate, we found that: (1) miR-18b-3p is the first circulatory miRNA biomarker predictive of radiation therapy/chemotherapy-induced OM; (2) miR-18-3p mechanistically promotes angiogenesis through SEMA3G/FOXJ2 selective targeting; (3) miR-18b-3p holds important translational significance on the prevention and treatment of OM. Being chemo- and radiotherapy still two mainstream treatments for cancer therapy, the implications of the reported findings might be of paramount importance to improve the quality of life of cancer patients.

## Supplementary information

Additional file

## Data Availability

Further information and requests for resources and reagents should be directed to and will be fulfilled by the lead contact, Giovanni Blandino (giovanni.blandino@ifo.it) and Sabrina Strano (sabrina.strano@ifo.it). Materials generated in this study will be available upon fulfilment of the material transfer agreement (MTA). Original/source data of RNA sequencing have been deposited at Zenodo database 10.5281/zenodo.17416581 and are publicly available as of the date of publication.
