# Supplementary material for: Circulating miR-18b-3p is a novel biomarker predicting chemo-radiotherapy induced oral mucositis in head and neck cancer
Source: Signal Transduct Target Ther. 2025 Nov 21;10:380. doi: 10.1038/s41392-025-02478-3 (PMC12635060; doi:10.1038/s41392-025-02478-3)
Supplement: Supplementary file 1 — Additional file [file 41392_2025_2478_MOESM1_ESM.docx]

**Material and Methods**

**Cell Lines and culture conditions:** Human Primary Gingival Epithelial Keratinocytes (EGK) (ATCC, PCS-200-014) and Primary Epidermal Keratinocytes (HEKa) (ATCC, PCS-200-011) cell lines were obtained from ATCC (Rockville, MD, USA) and cultured in dermal cell basal medium (ATCC, PCS-200-030) supplemented with keratinocyte growth kit (PCS-200-040; ATCC), and 0.1% of penicillin (100 U/mL) and streptomycin (100 mg/mL; Invitrogen-GIBCO). Immortalized Human Oral Gingival Keratinocytes (HPV-16 GM) (IGK) (ABM, T0717) were purchased from Applied Biological Materials Inc. (abm; Richmond, BC, Canada) and cultured in Prigrow X medium (ABM, TM0717) supplemented with 0.1% of penicillin (100 U/mL) and streptomycin (100 mg/mL; Invitrogen-GIBCO). Human umbilical vein endothelial cells (HUVEC) were purchased from Lonza (Switzerland) and were cultured in endothelial cell growth basal medium 2 (EBM-2, CC-3156, Lonza) supplemented with growth factors and Single Quots TM (CC-4176, Lonza).

**Treatments:** Cisplatin (Selleckchem, S1166), oxaliplatin (Selleckchem, S1224), ifosfamide (Selleckchem, S1302), capecitabine (Selleckchem, S1155), azacytidine (Selleckchem, S1782), 5-fluorouracil (Selleckchem, S1209), irinotecan (Selleckchem, S1198), erlotinib (Selleckchem, S7786), everolimus (Selleckchem, S1120), cetuximab (Selleckchem, A2000), alpelisib (BYL719) (Selleckchem, S2814), paclitaxel (Selleckchem, S1150), docetaxel (Selleckchem, S1148), vinolrebine (Selleckchem, S4269), doxorubicin (Selleckchem, S1208), all drugs were dissolved according to the manufacturer’s instructions at the final concentration of 10 mM. Briefly, 2x10^5^cells/well were plated in 96 well, then, after 24 hours cells were treated with different doses of drug for 72 hours.

Cells were seeded in 60 mm^2^ dishes and after 24 hrs were irradiated at 1,2 and 4Gy with the IBL 437 C irradiator according to the manufacturer’s instructions.

**Endothelial tube formation assay:** HUVEC (2.5 X 10^4^) were seeded in a 96-well culture plate precoated with 50 μl/well of growth factor reduced matrix (Cultrex, R&D Systems, USA). The day after, cells were stimulated with serum-free medium or CM derived from EGK or IGK cells previously treated or not with cisplatin for 72 h. Alternatively, HUVEC (1.6 X10^5^) were seeded in a 48-well culture plate precoated with 200 μl/well of Cultrex and transfected with 2nM of mirVana miRNA mimic-hsa-miR-18b-3p or with the negative miRNA control #1 (Ctr-mimic-miR), or stimulated with VEGF (50 ng/ml, R R&D Systems). Cells were left overnight at 37 °C. Capillary-like structure were captured at 20X of magnification with a ZOE Fluorescent Cell Imager (BioRad Laboratories, USA). Tube formation was analyzed by using the Angiogenesis Analyzer for ImageJ software measuring the number of segments and the tube length.

**Cell Transfection and transactivation assays:** [Lipofectamine](https://www.sciencedirect.com/topics/medicine-and-dentistry/lipofectamine) RNAimax (Invitrogen) was used according to the manufacturer's instruction for transfection with miR-18b-3p [miRNA](https://www.sciencedirect.com/topics/medicine-and-dentistry/microrna) mimics or siRNA for FOXJ2 (sc-62337, Santa Cruz) or for SEMA3G (sc-78531, Santa Cruz). The mirVana™ miRNA Mimic miR-18b-3p was used at the final amount of 2nM. The mirVana™ miRNA Mimic Negative Control #1 (Ambion) was used as control of transfection. The final concentration of 100 pmol was used for silenced FOXJ2 or SEMA3G in HUVEC cells. The pmirNanoGlo-SEMA3G Fusion and the pmirNanoGlo-FOXJ2 Fusion vectors (Promega) were used to assess the binding between the 3’UTR of either SEMA3G or FOXJ2 and miR-18b-3p. Lipofectamine 2000 (Invitrogen) was used to transfect the cells with the miR-18b-3p mimic and with the mutated (Eurofins) or the wildtype plasmids for FOXJ2-3UTR and SEMA3G-3UTR. For each condition, 100 ng of plasmid was used.

**RNA extraction and expression analysis:** total RNA was extracted using the Trizol reagent (ThermoFisher Scientific, USA), according to the indications of the manufacturer. RNA concentration and purity were determined with a Nanodrop 1000 (ThermoFisher Scientific). cDNA was synthesized according to the manufacturer’s instructions (M-MLV RT kit, Invitrogen). Gene expression was measured by real-time PCR using the FastStart SYBR Green Master nMix (Applied Biosytems) on a QuantStudio 6-Flex (ThermoFisher Scientific), according to the manufacturer’s instruction. (Applied Biosystems). Sequences of qPCR primers are reported in supplementary table Final data were obtained by using the 2−ΔΔCt method.

**Patients’ samples:** Sample patients derived from HNSCC (n=23) cancer patients treated with radio-chemotherapy

| **sex** | **age** | **tumor site** | **smoke** | **alcohol** | **TNM** | | | **Rtdos** | **CT** | **mucositis** | |
| --- | --- | --- | --- | --- | --- | --- | --- | --- | --- | --- | --- |
|  |  |  |  |  | **cT stage** | **cNstage** | **cM** |  |  | **1 month** | **2 months** |
| M | 75 | oral cavity | 3 | 3 | 2 | N2b | 0 | 60GY |  | G2 | N.A. |
| M | 73 | pyriform sinus | 0 | 1 | 4a | N2c | 0 | 60GY |  | 0 | N.A. |
| M | 72 | pyriform sinus | 3 | 2 | 4a | 3b | 0 | 60GY | x | 0 | N.A. |
| M | 58 | Larynx | 3 | 2 | 4 | 3b | 0 | 60GY | x | G2 | 0 |
| F | 60 | palatine tonsil | 3 | 1 | 4 | 1 | 0 | 70GY | x | G3 | G3 |
| M | 63 | Tonsil | 0 | 2 | 4 | N1 | 0 | 70GY | x | G2 | G2 |
| M | 59 | palatine tonsil | 0 | 2 | 4a | 3b | 0 | 70GY | x | G2 | G2 |
| F | 64 | base of the tongue | 0 | 1 | 4 | N1 | 1 | 70GY | x | 0 | G2 |
| M | 64 | base of the tongue | 3 | 1 | 4a | N2b | 0 | 60GY | x | G1 | G1 |
| M | 58 | Orophariynx | 3 | 2 | T2 | N3b | 0 | 70GY | x | G2 | G2 |
| M | 55 | palatine tonsil | 3 | 2 | no | no | 0 | 70GY | x | 0 | G2 |
| M | 73 | oro supraglottic larynx | 3 | 2 | 4 | N2 | 0 | 70GY | x | G2 | G2 |
| M | 60 | base of the tongue | 0 | 1 | 1 | N3 | 0 | 70GY | x | G2 | G2 |
| M | 68 | upper larynx | 3 | 1 | 3 | N2c | 0 | 70GY | x | G2 | 0 |
| M | 81 | palatine tonsil | 3 | 2 | 3 | N1 | 0 | 70GY | x | G2 | G2 |
| M | 48 | Rinopharynx | 2 | 2 | 1 | N1 | 0 | 70GY | x | 0 | G1 |
| M | 61 | palatine tonsil | 3 | 2 | 3 | N0 | 0 | 70GY | x | G2 | G2 |
| F | 78 | Orophariynx | 0 | 2 | 4 | 2 | 0 | 70GY | x | G2 | G2 |
| M | 58 | Rinopharynx | 1 | 1 | 2 | N2 | 0 | 70GY | x | 0 | G1 |
| F | 46 | base of the tongue | 0 | 1 | 4 | N2 | 0 | 70GY | x | 0 | G2 |
| M | 58 | Tonsil | 2 | 1 | x | N2 | 0 | 70GY | x | G1 | G2 |
| M | 69 | base of the tongue | 0 | 1 | 2 | N3b | 0 | 70GY | x | G2 | G2 |
| M | 77 | base of the tongue | 0 | 0 | 4a | N2 | 0 | 70GY | N.A. | G1 | G1 |

and from HNSCC patients treated with the SBRT-CT regimen (n=10) were recruited at the Regina Elena Cancer Institute.

| **sex** | **age** | **tumor site** | **smoke** | **alcohol** | **TNM** | | | **Rtdos** | **CT** | **mucositis** |
| --- | --- | --- | --- | --- | --- | --- | --- | --- | --- | --- |
|  |  |  |  |  | **cT stage** | **cNstage** | **cM** |  |  |  |
| M | 68 | Glottic ca | current | no | 1a | 0 | 0 | 36/3 | no | 0 |
| M | 66 | Glottic ca | current | social | 1b | 0 | 0 | 36/3 | no | 0 |
| M | 60 | Glottic ca | previous | social | 1b | 0 | 0 | 36/3 | no | 0 |
| M | 73 | Glottic ca | current | heavy | 1a | 0 | 0 | 36/3 | no | 0 |
| F | 64 | Glottic ca | current | heavy | 1a | 0 | 0 | 36/3 | no | 0 |
| M | 62 | Glottic ca | current | social | 1a | 0 | 0 | 36/3 | no | 0 |
| F | 55 | Glottic ca | previous | no | 1a | 0 | 0 | 36/3 | no | 0 |
| F | 58 | Glottic ca | current | no | 1a | 0 | 0 | 36/3 | no | 0 |
| F | 80 | Glottic ca | no | no | 1a | 0 | 0 | 36/3 | no | 0 |
| M | 71 | Glottic ca | previous | no | 1b | 0 | 0 | 36/3 | no | 0 |

Circulating miR-18b-3p was validated “in vivo” by collecting plasma samples from cancer patients treated with different therapeutic regimens and affected or not of oral mucositis of different grading. The respective clinical study protocols were approved by Ethical Committee of National Cancer Institute, Roma. Plasma was isolated by centrifugation at 1200 x g at 4°C for 10 minutes followed by a centrifugation at 12000 rpm at 4°C for 10 minutes. Supernatants were carefully transferred into new RNAse/DNAse free cryogenic vials and stored at -80° C until use.

**Plasma and media RNA extraction and qPCR:** total RNA from plasma specimen or cell culture media was extracted by the Magmax Total RNA isolation kit (Thermofisher), according to the manufacturer’s instruction. The first-strand cDNA was synthesized according to the manufacturer’s instructions (TaqMan advanced miRNA cDNA synthesis kit, ThermoFisher Scientific). qRT-PCR was performed with Luna Universal Probe qPCR Master Mix (New England Biolabs, USA) on QuantStudio 5 (ThermoFisher Scientific). The advanced taqman assay (ThermoFisher Scientific) probe for miR-18b-3p was used. The C. Elegans miR-39 (Serum/Plasma Spike-In Control, ThermoFisher Scientific) was used as control to standardize the miRNA expression.

**RNA processing and hybridization:** Total RNA from media of human gingival keratinocytes was extracted by miRNeasy Serum/Plasma advanced kit, according to manufacturer's instructions. Agilent's microRNA Complete Labeling and Hyb Kit (Agilent) was used to generate fluorescent microRNA, according to manufacturer's instructions. Scanning and image analysis were performed using the Agilent DNA Microarray Scanner (P/N G2565BA). Feature Extraction Software (V-10.5) was used for data extraction from raw microarray image files.

**RNA sequencing (RNA-seq) analysis:** for RNA seq, through next generation sequencing, we extracted RNA from HUVEC cells transfected with mimic CTRL or mimic miR-18b-3p for 48 hours using miRNeasy kit (Qiagen) following the manufacturer’s instructions. RNAseq of total RNA from n=4 separate replicates of both mimic CTRL and mimic 18b-3p was performed on and Illumina HiSeq 2500 platform according to the following parameters: Hiseq2500 4 plex run, 2 × 100 bp reads, about 60 M reads/sample (for mRNAs/lincRNAs) after preparing the libraries with the Truseq stranded with RiboZero Illumina kit. RNA-seq data were analyzed with “rnaseq” version 3.9 pipeline included in the nf-core platform (<https://nf-co.re/rnaseq/3.9>) using default parameters. The alignment was performed using Star to map the FastQ reads to the reference genome and perform downstream BAM-level quantification with Salmon Normalized TPM values were used to determinate gene modulation among different condition. A hierarchical clustering analysis was conducted to illustrate differences in gene expression between conditions. Sample distances were calculated using the Euclidean distance metric and average linkage method. Genes with an adjusted p-value < 0.05 from DESeq analysis were selected as statistically significant. The analyses were performed using MATLAB R2023a software.
